# Comparative efficacy and safety of intravenous versus nebulized magnesium sulfate for acute asthma exacerbations: an overview of systematic reviews and meta-analyses

**DOI:** 10.3389/fmed.2026.1917060

**Published:** 2026-08-14

**Authors:** Yongji Wang, Zhuang Wang, Shengtao Cui, Lijie Hou, Bing Tian, Xiaofei Xie, Yongfu Song, Shulei Liu, Ying Wang

**Affiliations:** 1 College of Traditional Chinese Medicine, Changchun University of Chinese Medicine, Changchun, Jilin, China; 2 Department of Pediatric Internal Medicine One, Affiliated Hospital of Changchun University of Chinese Medicine, Changchun, Jilin, China

**Keywords:** asthma, intravenous, magnesium sulfate, nebulized, overview of systematic reviews and meta analyses

## Abstract

**Objective:**

This study aimed to conduct an umbrella review comparing intravenous versus nebulized magnesium sulfate for acute asthma exacerbations, providing evidence to guide route selection and clinical decision-making.

**Methods:**

We systematically searched PubMed, Embase, Cochrane Library, Web of Science, CNKI, VIP, WANFANG, and CBM from inception to August 2025, for systematic reviews/meta analyses comparing intravenous versus nebulized magnesium sulfate for acute asthma exacerbations. Overlap among primary studies was assessed using a citation matrix and corrected covered area. Quality assessment was performed using ROBIS, AMSTAR-2, PRISMA 2020, and GRADE. Quantitative and qualitative analyses of primary outcomes were performed.

**Results:**

A total of 18 systematic reviews/meta analyses were included. The corrected covered area was 5.168%, indicating some overlap in primary studies. ROBIS judged all studies at low risk of bias. AMSTAR-2 rated 6 studies as high quality, 2 as moderate, and 10 as low. PRISMA 2020 scores ranged from 25.5 to 41, with 10 studies of high and 8 of moderate reporting quality. GRADE yielded 13 high-quality, 38 moderate-quality, 27 low-quality, and 26 extremely low-quality outcomes.

**Conclusion:**

This overview clarifies the role of intravenous magnesium sulfate in acute asthma: not for routine use but as secondary adjunctive therapy for severe cases refractory to first-line treatment. The role of nebulized magnesium sulfate is more complex—neither universally applicable nor a replacement for first-line medications. Current evidence does not support routine use, warranting only a weak recommendation for patients who fail standard therapy. Future research should focus on its synergistic effects with other therapies and further elucidate its mechanisms.

**Systematic review registration:**

https://www.crd.york.ac.uk/PROSPERO/, PROSPERO: CRD420251122150.

## 1 Introduction

Bronchial asthma, a highly heterogeneous chronic airway inflammatory disease involving multiple cells and cellular components (1, 2), affects nearly 300 million people worldwide (3). While the incidence of asthma has plateaued in economically developed regions, it continues to rise in less developed areas (4). Similarly, asthma mortality rates vary significantly across the globe, with higher rates observed in countries with underdeveloped healthcare systems (4–6). This situation has emerged as a new public health challenge (7, 8). The substantial disparities in disease burden between regions underscore the necessity of optimizing existing interventions for asthma exacerbations and highlight the urgency of exploring combination therapies.

Magnesium sulfate (MgSO_4_) is a therapeutic agent used in clinical settings such as emergency departments and inpatient care for the management of acute severe asthma and status asthmaticus (9–11). Within the standardized clinical management framework for asthma, it is not classified as a conventional asthma controller medication (9). Its clinical role is defined as a “rescue” or “add-on” therapy sequentially administered when first-line conventional treatments (e.g., inhaled *β*₂-agonists and systemic corticosteroids) fail to achieve the desired therapeutic response (12–15). In the clinical application of MgSO_4_ for asthma, the primary routes of administration are intravenous injection and nebulized inhalation (16, 17). Numerous clinical studies have demonstrated that both intravenous and nebulized MgSO_4_ confer certain therapeutic benefits in asthma management (18–22). Recent systematic reviews/meta-analyses (SRs/MAs), particularly those published in 2024–2025, show marked disagreement and fragmented evidence. Outcome coverage is narrow, populations are poorly represented, and data on critical endpoints and long-term prognosis are scarce. Adult and pediatric studies are typically separated, precluding cross-population and cross-route synthesis. Moreover, existing reviews have not systematically assessed methodological quality, publication overlap, or evidence grading across all prior meta-analyses, making it difficult to establish a standardized evidence framework to differentiate target populations and recommendation levels for each route.

## 2 Materials and methods

### 2.1 Study design

This umbrella review addresses the methodological limitations of existing SRs/MAs, including narrow research perspectives, fragmented approaches, and reliance on narrative synthesis that precludes valid indirect comparisons. Given the continuous accumulation of primary evidence, effect estimates from prior reviews are subject to temporal bias. To overcome these issues, this study moves beyond the conventional umbrella review model of merely synthesizing published conclusions, and instead re-extracts original randomized controlled trial data within a unified framework to perform standardized quantitative meta-analysis. Quantitative re-synthesis and narrative summary are not mutually exclusive but complementary strategies for evidence integration. This study frames its research question and eligibility criteria around indirect comparisons between intravenous and nebulized magnesium sulfate administration routes, strictly adhering to the PICOS framework throughout. The details are as follows:

(1) Population: patients with acute asthma exacerbation, irrespective of severity; (2) Intervention: magnesium sulfate administered via intravenous or nebulized route, either alone or as add-on to standard care; (3) Comparator: placebo or standard care; (4) Outcomes: primary outcomes including admission rate, pulmonary function, vital signs, and adverse events; (5) Study design: SRs/MAs of randomized controlled trials (RCTs) only.

### 2.2 Protocol registration

The study protocol was prospectively registered in the PROSPERO database (Registration ID: CRD420251122150) prior to study initiation, with the registration date preceding the commencement of literature retrieval. The actual implementation process of this study did not deviate from the registered study protocol.

### 2.3 Inclusion criteria

(1) SRs/MAs in which MgSO_4_ serves as a primary or adjunctive intervention for the treatment of asthma; (2) The experimental groups receive interventions based on MgSO_4_ alone, MgSO_4_ combined with standard of care (SOC), or MgSO_4_ with placebo, while the control groups receive either placebo or SOC; (3) All primary studies included in the eligible SRs/MAs are RCTs; (4) The route of administration is restricted to intravenous injection or nebulized inhalation; (5) Studies employing a single-blind, double-blind, or open-label design where participants and/or investigators, or both, are unaware of the assignment to MgSO_4_, SOC, or placebo; (6) Studies in which participants are allocated to either the experimental or control group via randomized or non-randomized methods.

### 2.4 Exclusion criteria

(1) Duplicate publications; (2) Publications for which the full text or complete data are unavailable; (3) Narrative reviews; (4) Studies deviating from the research theme; (5) Primary clinical studies; (6) Animal studies; (7) Research on content homogeneity.

### 2.5 Search strategy

Search all SRs/MAs literature on the treatment of asthma with MgSO_4_ from the establishment of the databases to 10 August 2025 in eight databases, namely PubMed, Embase, Cochrane Library, Web of Science, CNKI, VIP, WANFANG, and CBM, through the computer network. The search terms and search strategy are as follows (taking Web of Science as an example):

#1:(((((((((((TS = (asthma)) OR TS = (bronchial hyperreactivity)) OR TS = (airway remodeling)) OR TS = (airway inflammation)) OR TS = (respiratory hypersensitivity)) OR TS = (allergic asthma)) OR TS = (Non-Allergic Asthma)) OR TS = (Exercise-Induced Asthma)) OR TS = (occupational asthma)) OR TS = (severe asthma)) OR TS = (childhood asthma)) OR TS = (asthmatic).

#2:((((((((TS = (Magnesium Sulfate)) OR TS = (Magnesium sulphate)) OR TS = (MgSO4)) OR TS = (MgSO₄)) OR TS = (Epsom Salt)) OR TS = (Epsom Salts)) OR TS = (Bitter salt)) OR TS = (Bitter salts)) OR TS = (Epsomite).

#3:(((TS = (meta)) OR TS = (meta-analysis)) OR TS = (meta-analysis)) OR TS = (systematic review).

#4:#1 AND #2 AND #3.

### 2.6 Literature screening and data extraction

This study adopted a dual-independent literature screening mechanism to ensure the rigor of the search results. Two systematically trained retrievers performed the systematic search in specified databases according to a predefined search strategy. Upon completion of the initial search, cross-verification was conducted to assess the consistency of the results. In cases of screening discrepancies, a consensus meeting was initiated to perform a root cause analysis, including dimensions such as the sensitivity of search terms and the applicability of inclusion criteria. If discrepancies persisted, a third senior researcher (associate professor or above) exercised arbitration authority to ultimately determine the final literature pool that met the predefined inclusion/exclusion criteria. The selected literature was required to be recorded using a standardized data extraction form, capturing the following raw data: (1) Bibliographic identification information (first author and publication year); (2) Study characteristics (study type, sample size, number of included studies); (3) Methodological elements (intervention/comparator schemes and quality assessment tools); (4) Key findings (primary outcome measures and core conclusions).

### 2.7 Extraction of repetition rate

In the evidence-based medicine research framework, SRs/MAs often lead to multiple inclusion of the same original studies due to systematic literature searching and pooled analysis, potentially causing distortion of evidence weighting. To quantify such overlap, a cross-reference matrix of original studies versus systematic reviews is constructed, with SRs/MAs as columns and all original RCTs as rows, marking how many meta-analyses each original study is included in, and the Corrected Covered Area (CCA) is calculated for precise measurement (23). This metric is computed using the formula CCA = (N - R) / (R × C - R), where N represents the total number of primary studies with duplicate counts, R denotes the number of independent primary studies after deduplication, and C refers to the number of included SRs/MAs. According to international consensus, the CCA value range is divided into four evidence overlap levels: ≤5% indicates acceptable slight overlap, 5–10% suggests moderate overlap requiring caution, 10–15% represents high overlap that may influence conclusions, and >15% signifies severe evidence redundancy. If moderate or higher overlap is identified, a combined quantitative and qualitative analysis strategy will be adopted to integrate outcome data from the included SRs/MAs.

### 2.8 Quality assessment

#### 2.8.1 Risk of bias assessment

The risk of bias in the included SRs/MAs was assessed using the Risk of Bias in Systematic Reviews (ROBIS) tool through a three-phase process (24). In the relevance verification phase, the alignment between the systematic review question and clinical practice needs was evaluated based on PICO elements, with tailored criteria applied to different study types such as interventional and diagnostic studies. We assessed process bias by rating 21 signaling questions across five procedural domains—search strategy, screening, extraction, quality appraisal, and synthesis. Each item was scored on a five-level scale (Yes, Probably Yes, No, Probably No, or No Information) to standardize the measurement of operational rigor. In the final phase, overall risk of bias was determined as low, high, or unclear based on synthesized evidence from the preceding stages.

#### 2.8.2 Methodological quality assessment

The measurement tool to assess systematic reviews 2 (AMSTAR-2) is a widely recognized and authoritative tool for evaluating the methodological quality of SRs/MAs (25, 26). Comprising 16 items, AMSTAR-2 designates items 2, 4, 7, 9, 11, 13, and 15 as critical, while the remaining items are classified as non-critical. Each item is rigorously assessed and rated as “Yes,” “No,” or “Partial Yes.” AMSTAR-2 rates methodological quality as high, moderate, low, or critically low by weighing both critical and non-critical item assessments and their respective deficiency counts. This rigorous grading system serves as an authoritative quality-assessment tool, enabling reliable evaluation of methodological validity for included studies.

#### 2.8.3 Reporting quality assessment

This study utilized the Preferred Reporting Items for Systematic Reviews and Meta Analyses 2020 (PRISMA 2020) statement as the core methodological tool to systematically evaluate the reporting quality of included studies (27, 28). The guideline establishes a standardized reporting framework covering seven domains: Title, Abstract, Introduction, Methods, Results, Discussion, and Supplementary Information. These domains are further divided into 27 main items and 42 sub-items, scored using a dichotomous system (Y/PY/N), with a maximum total score of 42 points. Quality grading was performed according to predefined criteria. High quality (33–42 points) represents ≥80% reporting completeness with fulfillment of most PRISMA requirements, demonstrating methodological rigor and result reproducibility. Moderate quality (25–32 points) reflects 60–79% compliance, where non-critical omissions—such as insufficiently described search strategies—do not substantially affect overall conclusions. Low quality (<25 points) falls below the 60% benchmark, indicating inadequate reporting of core methodological or results components, including risk of bias assessment or data synthesis procedures, which may potentially introduce conclusion bias.

#### 2.8.4 Evidence quality assessment

The Grading of Recommendations Assessment, Development and Evaluation (GRADE) system, recognized internationally as the standard for assessing evidence quality (29), enables multidimensional evidence classification through systematic evaluation of evidence bodies. Its core assessment domains include: limitations in study design (risk of bias), consistency of results (heterogeneity), directness of evidence (applicability to population/intervention/outcomes), precision of effect estimates (width of confidence intervals), and publication bias potential. Through integrated analysis of these elements, the certainty of evidence is categorized into four levels: “high,” “moderate,” “low,” or “very low”.

### 2.9 Quantitative analysis

This study performed quantitative data synthesis of the included SRs/MAs, with all pooled analyses based on individual patient data re-extracted from the original randomized controlled trials, rather than directly reusing the published summary effect sizes from the included meta analyses. Pooling was conducted on the premise that the outcome definitions, measurement methods, and effect sizes of the original studies were clinically homogeneous or amenable to methodological standardization. Effect sizes were calculated by variable type: relative risk (RR) for dichotomous variables and standardized mean difference (SMD) for continuous variables, both reported with point estimates and 95% confidence intervals (95% CIs). Heterogeneity was assessed using the *I*^2^ statistic and corresponding *p*-values: fixed-effects models were applied when *I*^2^ ≤ 50% and *p* > 0.1; otherwise, random-effects models were used. In cases of significant heterogeneity (*I*^2^ > 50% and *p* ≤ 0.1), sensitivity and subgroup analyses were conducted to identify sources, thereby reaching conclusions that balance statistical power and clinical consistency. Effect estimates for intravenous and nebulized administration were derived independently; between-group differences were interpreted only as indirect comparisons without head-to-head randomized controlled trial evidence, serving solely to describe numerical trends and not to confirm genuine efficacy differences between routes of administration.

All meta-analyses were performed using the meta-analysis module on the SPSSAU online platform.^1^ Heterogeneity was assessed by Q-test *p*-value, *I*^2^ statistic, and H value. To explore sources of heterogeneity, subgroup analyses were prespecified based on study region (or the actual grouping variable used), with the subgroup variable entered into the “Subgroup” column of SPSSAU; the system automatically performed within-group pooling and between-group comparisons, with interaction tested by Q-test (*p* < 0.05 considered significant). Sensitivity analysis used the leave-one-out method via the “sensitivity test” option in SPSSAU, sequentially excluding each study and re-running the meta-analysis; robustness was considered adequate if the direction and statistical significance (95% CI) of the pooled effect did not fundamentally change (i.e., no reversal of conclusions) after any single exclusion. Publication bias was evaluated using funnel plots combined with Begg’s rank correlation test and Egger’s linear regression test; *p* > 0.05 for either test and roughly symmetric funnel plots indicated no significant publication bias, with trim-and-fill applied when necessary to correct the pooled effect estimate.

### 2.10 Qualitative analysis

For outcome measures unsuitable for quantitative analysis in the included SRs/MAs, qualitative analysis methods were employed. Adopting an integrative approach combining narrative synthesis and critical appraisal, we applied thematic analysis to accurately extract key themes from textual descriptions and graphical interpretations of study results. The main findings regarding outcome measures across studies were systematically synthesized, with common conclusions and contradictory findings clearly delineated. Based on this synthesis, feasible directions for future research were proposed, thereby providing comprehensive and in-depth qualitative evidence for subsequent decision-making.

## 3 Results

### 3.1 Results of literature screening

**Figure 1:**
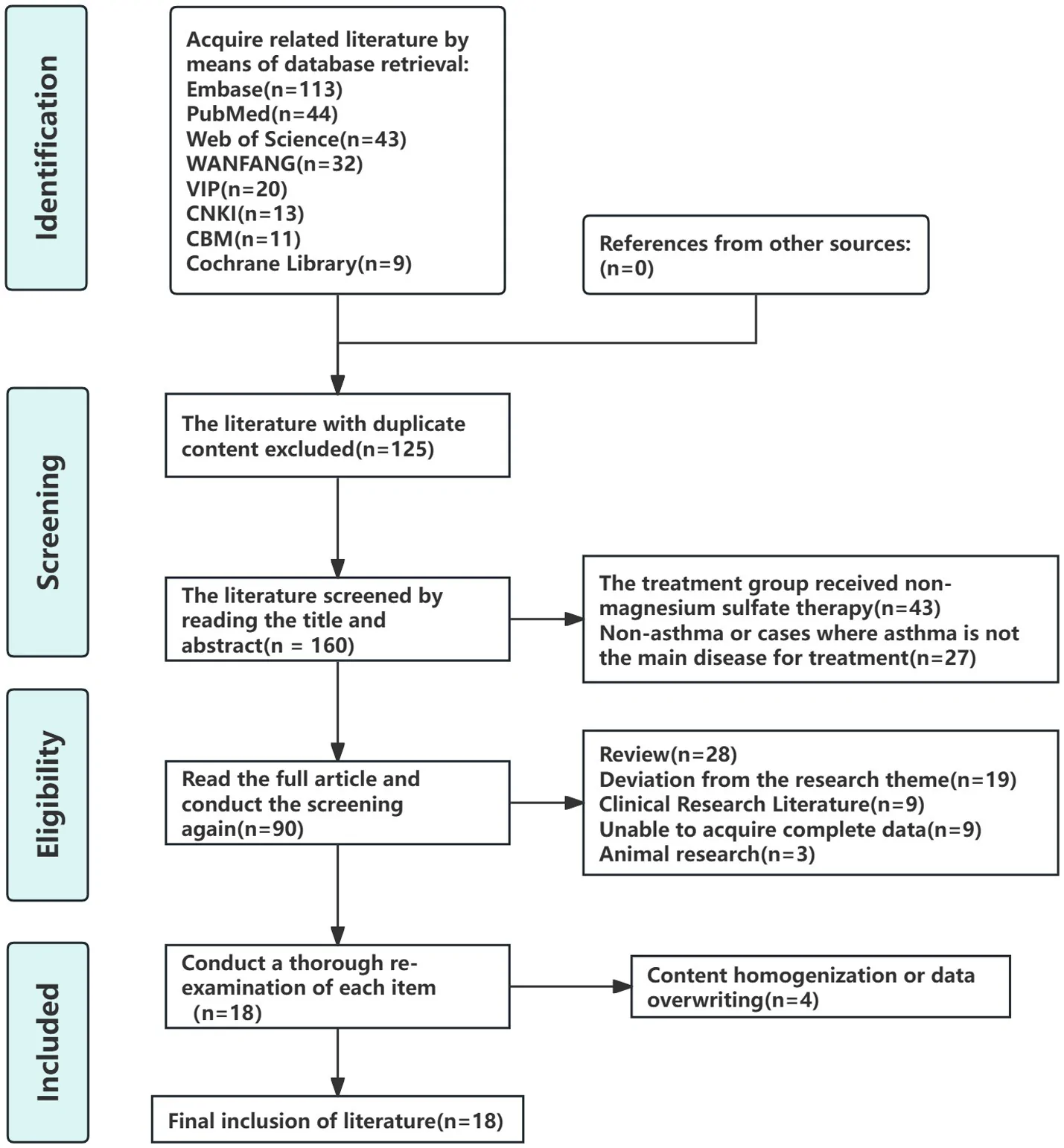
Literature screening process.

The initial search identified 285 publications. After removing 160 duplicates using EndNote X9, 125 records underwent title and abstract screening, which led to the exclusion of 43 articles that did not focus on asthma or where asthma was not the primary disease under treatment, and 27 articles with intervention regimens in the treatment or control groups that did not meet the eligibility criteria. Following full-text assessment of the remaining 55 articles, 28 reviews, 19 articles with deviating research topics, 9 clinical studies, 9 publications with incomplete data, and 3 animal studies were excluded. A comprehensive re-evaluation of the remaining 22 articles was performed, resulting in the exclusion of 4 studies due to substantial content overlap. Ultimately, 18 articles were included (30–47). The study selection process is illustrated in Figure 1.

### 3.2 Basic characteristics of the included literature

The final analysis included 13 English and 5 Chinese publications, comprising 1 dissertation and 17 journal articles published between 2000 and 2025. Across the 18 included studies, the intervention group received MgSO_4_ (with or without SOC), while the control group received placebo (with or without SOC). Regarding outcome measures, all 18 studies reported pulmonary function; 14 studies reported admission to hospital and adverse outcomes and side effects; 10 studies reported vital signs and clinical symptom scores; 7 studies reported treatment time or duration of symptoms; 5 studies reported intensive care admission; 3 studies reported recurrence rate; 2 studies reported overall response rate; and individual studies reported remission rate of clinical signs, school absence in days, and need for invasive mechanical ventilation or non-invasive ventilation. For quality assessment, 6 studies applied the JADAD scale, while 14 utilized the Cochrane Risk of Bias tool. Regarding the study conclusions, 9 studies indicated that heterogeneity among the included trials precluded more definitive conclusions, and conflicting findings were observed across studies. The basic characteristics of the included studies are summarized in Table 1.

**Table 1:** Characteristics of the included literature.

First author and year	Number of literatures/Sample size	The types of original documents	Intervention measures		Bias Risk Measurement Tool	Route of Administration	Endpoint measure	The main conclusions of the author
			Treatment group	Control group				
Rowe et al. (30)	7/665	RCT	MgSO_4_ + SOC	SOC + Placebo	Cochrane, JADAD	Intravenous	①②③④⑤	(1)(2)(3)
Rowe et al. (31)	7/668	RCT	MgSO_4_ + SOC	SOC + Placebo	JADAD	Intravenous	①②③④	(1)(2)
Cheuk et al. (32)	6/296	RCT	MgSO_4_ + SOC	SOC + Placebo	Cochrane, JADAD	Intravenous	①②⑥	(5)
Blitz et al. (33)	5/219	RCT	MgSO_4_ + SOC	SOC + Placebo	JADAD	Nebulized	①②③	(6)(7)(8)
Lu and Zhou (34)	24/1669	RCT	MgSO_4_ + SOC	SOC + Placebo	JADAD	Nebulized	①②③	(6)
Powell et al. (35)	16/896	RCT	MgSO_4_ + SOC	SOC + Placebo	Cochrane	Nebulized	①②③④⑤⑥	(6)(8)(9)(10)
Kew et al. (36)	25/1754	RCT	MgSO_4_	Placebo	JADAD	Intravenous	①②③④⑤⑥⑦	(3)(8)
Donghai Wang (37)	9/844	RCT	MgSO_4_ + SOC	SOC + Placebo	JADAD	Nebulized	①②④⑥	(9)
Ma and Zhao 2016 (38)	8/1161	RCT	MgSO_4_ + SOC	Placebo	Cochrane	Intravenous	②④⑤⑧⑨	(3)(4)(8)
Ling et al. (39)	12/1992	RCT	MgSO_4_	Placebo	Cochrane	Nebulized	①②④	(10)
Knightly et al. (40)	25/2907	RCT	MgSO_4_ + SOC	SOC + Placebo	Cochrane	Nebulized	①②③④⑥⑦⑧	(8)(10)(11)(12)
Kassab et al. (41)	8/1585	RCT	MgSO_4_ + SOC	SOC	Cochrane	Nebulized	②③⑥	(9)
Zhang et al. (42)	14/1198	RCT	MgSO_4_ + SOC	SOC + Placebo	Cochrane	Nebulized	②④⑩	(6)(11)
Ambrozej et al. (43)	11/672	RCT	MgSO_4_ + SOC	SOC + Placebo	Cochrane	Intravenous	①②④⑤⑥⑦⑧	(2)(3)(8)
Kumar et al. (44)	10/2301	RCT	MgSO_4_ + SOC	SOC	Cochrane	Nebulized	①②③④⑤⑥⑦	(8)(10)
et al. Cunha (45)	12/2484	RCT	MgSO_4_ + SOC	SOC + Placebo	Cochrane	Nebulized	①②③④⑥⑪	(6)
et al. Zhong (46)	16/2601	RCT	MgSO_4_ + SOC	SOC + Placebo	Cochrane	Intravenous	②④⑩	(2)(8)
Hamud et al. (47)	9/473	RCT	MgSO_4_ + SOC	SOC + Placebo	Cochrane	Intravenous	①②④⑤⑥⑦⑫⑬	(2)(3)(8)(13)

① Admission to hospital. ② Pulmonary function. ③ Vital signs. ④ Adverse Outcomes and Side Effects. ⑤ Treatment time or Duration of Symptoms. ⑥ Clinical symptom score. ⑦ Intensive Care. ⑧ Recurrence rate. ⑨ Remission rate of clinical signs. ⑩ Overall response rate. ⑪ Requirement for intravenous bronchodilator therapy. ⑫ School Absence in Days. ⑬ Need for invasive mechanical ventilation or non-invasive ventilation. (1) Routine intravenous MgSO_4_ is not recommended for all acute asthma patients in the emergency department. (2) Intravenous MgSO_4_ has a favorable safety profile and demonstrated efficacy in severe acute asthma. (3) Intravenous MgSO_4_ reduces admission rates and improves pulmonary function. (4) Intravenous MgSO_4_ combined with montelukast for asthma increases the incidence of adverse drug reactions. (5) The addition of intravenous MgSO_4_ to standard therapy may provide benefit in children with moderate to severe acute asthma. (6) Nebulized MgSO_4_ combined with a β₂-agonist improves pulmonary function in patients with acute asthma exacerbation. (7) Nebulized MgSO_4_ combined with a β₂-agonist reduces the risk of admission in patients with acute asthma exacerbation. (8) Heterogeneity among the included trials precludes more definitive conclusions. (9) Nebulized MgSO_4_ cannot replace β₂-agonists. (10) The addition of nebulized MgSO_4_ to conventional therapy confers no clear benefit or only modest benefit overall. (11) Nebulized MgSO_4_ does not increase the risk of severe adverse reactions. (12) The benefits of nebulized MgSO_4_ may be more pronounced in patients with severe illness and short disease duration. (13) Intravenous MgSO_4_ reduces the need for non-invasive ventilation but has no significant effect on asthma scores, PICU admission, invasive ventilation, or length of hospital stay.

### 3.3 Duplication rate of the original literature

This study included a total of 18 SRs/MAs, encompassing 201 original studies. After removing duplicates, 104 unique studies remained. According to the formula, the CCA was calculated as (201–104) / (104 × 18–104) ≈ 0.05168, indicating a moderate degree of overlap. This reflects a moderate level of redundancy among the original studies included in the SRs/MAs.

### 3.4 Results of the risk of bias assessment

Of all the included studies (30–47), all successfully passed Phase 1 (applicability assessment) of the ROBIS tool. During Phase 2 assessment, across three key domains—study eligibility criteria, literature search, and synthesis methods—all studies (30–47) demonstrated low risk of bias. In the data quality domain of Phase 2, 17 studies (30–45, 47) were clearly judged as having low risk of bias, indicating high data reliability and quality. However, one study (46) was rated as “unclear” in terms of bias risk due to incomplete information on bias risk provided in the original research, which hindered precise determination of the accuracy of data extraction and evaluation. In Phase 3 (overall risk of bias), all studies (30–47) were assessed as having low risk of bias, with no high or unclear risk identified.

### 3.5 Results of the methodological quality assessment

**Figure 2:**
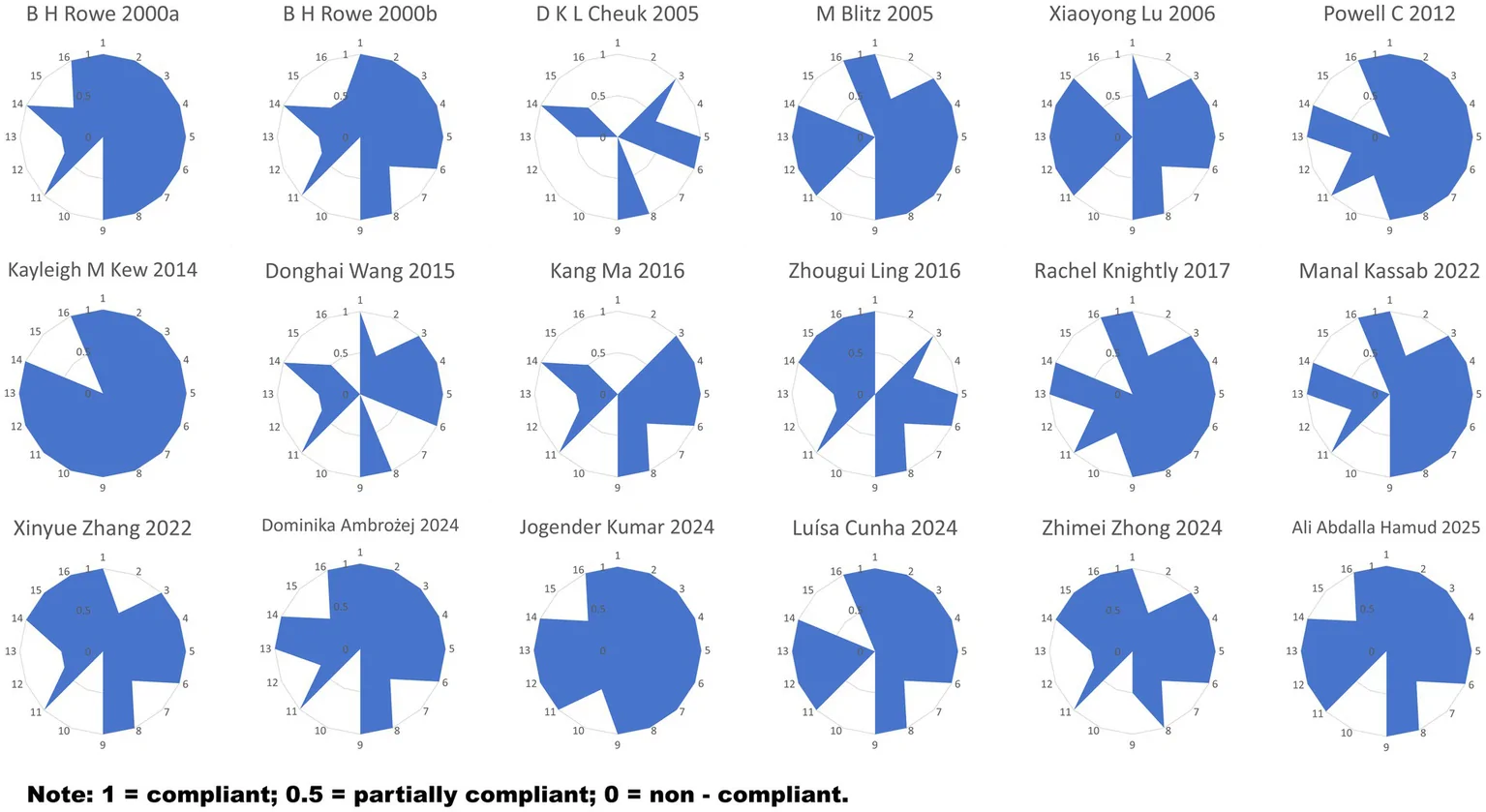
Radar chart of scores for each item of AMSTAR-2.

Among the included SRs/MAs, six studies were rated as high quality (30, 34, 42–44, 47), two as moderate quality (31, 46), and 10 as low quality (32, 33, 35–41, 45). For the key items, all 18 studies fully reported Item 11. The compliance rates for other key items were as follows: Item 9 (17/94.44%), Item 4 (16/88.89%), Item 13 (10/55.56%), Item 2 (8/44.44%), Item 7 (7/38.89%), and Item 15 (3/16.67%). Regarding non-key items, all 18 studies fully reported Items 1, 3, 5, 6, 8, and 14. The compliance rates for the remaining non-key items were: Item 16 (13/72.22%), Item 12 (6/33.33%), and Item 10 (1/5.56%). A detailed methodological quality assessment of the included literature is provided in Figure 2.

### 3.6 Results of the reporting quality assessment

**Figure 3:**
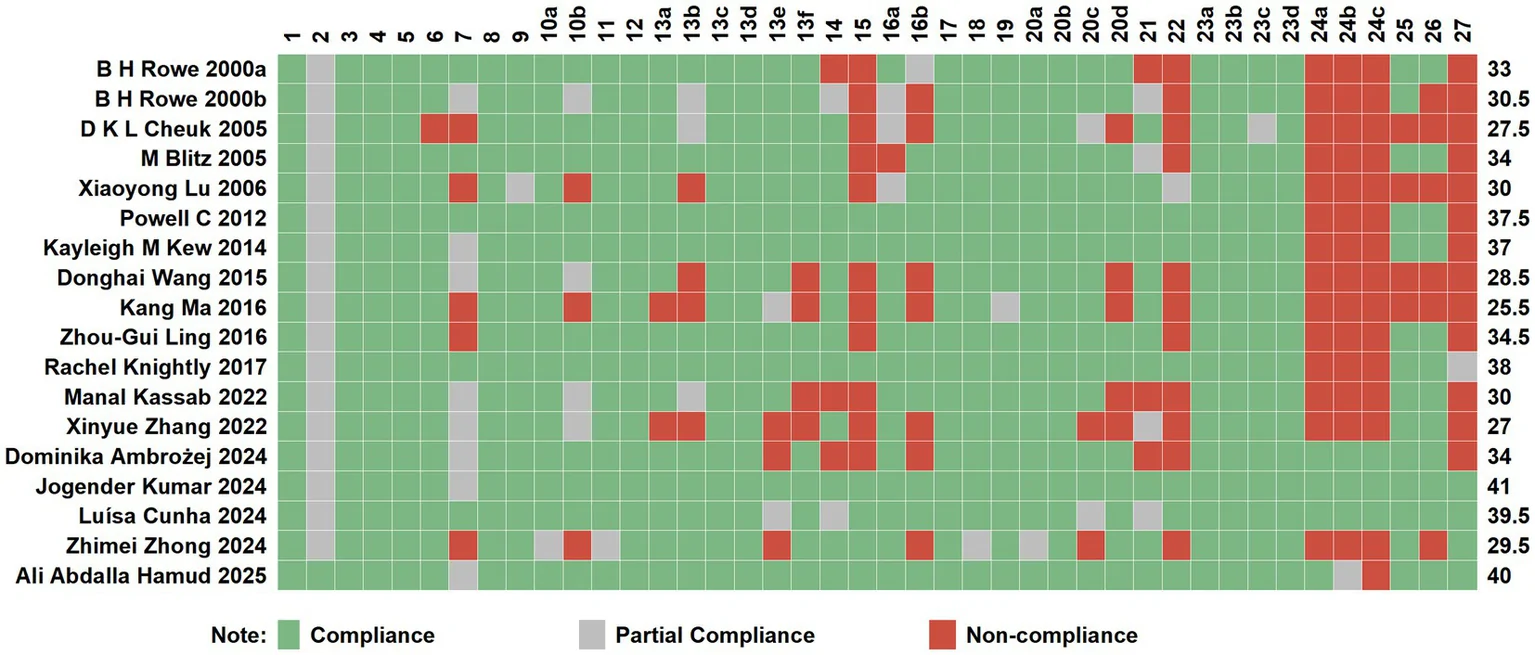
Cartesian heatmap of the scores of each item in PRISMA 2020.

The PRISMA 2020 checklist has a maximum score of 42 points, providing detailed criteria for assessing the completeness of reporting across sections including the abstract, introduction, methods, results, and discussion. The included studies achieved PRISMA 2020 scores ranging from 25.5 to 41 (mean 33.17). Of these, 10 articles were rated as high quality and eight as moderate quality. Regarding the 42 items, six items—specifically 15, 22, 24a, 24b, 24c, and 27—were fully reported in ≤50% of the 18 included studies, indicating substantial reporting deficiencies. These items relate to certainty assessment, certainty of evidence, registration and protocol, and availability of data, code, and other materials. Detailed results are presented in Figure 3.

### 3.7 Results of the evidence quality assessment

The GRADE approach was used to assess the quality of evidence for pooled outcome measures across the included studies. A total of 104 pooled effect estimates were evaluated. According to the results, 13 were rated as high quality, 38 as moderate quality, 27 as low quality, and 26 as very low quality. Detailed findings are presented in Table 2.

**Table 2:** Evidence quality assessment.

The included studies	Endpoint measure	Downgrading factor					Effect size	95% CI	*I*^2^/%	*p*	Evidence quality
		RB	IC	ID	IP	PB					
Rowe et al. (30)	Admission to hospital	−1^①^	−1^②^	0	−1^④^	−1^⑤^	OR = 0.31	[0.09,1.02]	76	0.013	Extremely low
	Peak Expiratory Flow Rate(PEFR)	0	−1^②^	0	−1^④^	0	WMD = 29.4	[−3.40,62.00]	59	0.079	Low
	Forced Expiratory Volume in 1 s(FEV1)%	0	−1^②^	0	−1^④^	0	WMD = 4.3	[−2.30,10.90]	73	0.20	Low
	Heart rate	0	−1^②^	0	−1^④^	0	WMD = 5.6	[−1.5,12.70]	87	0.72	Low
	Respiratory rate	0	0	0	−1^④^	0	WMD = -0.28	[−1.44,0.88]	43	0.63	Medium
	Blood pressure	0	−1^②^	0	−1^④^	0	WMD = -2.33	[−6.99,2.53]	59	0.36	Low
	Emergency Department Treatment Time	0	0	0	−1^④^	0	WMD = -5.28	[−9.34,−1.21]	0	0.011	Medium
Rowe et al. (31)	Admission to hospital	0	−1^②^	0	−1^④^	0	OR = 0.40	[0.15,1.07]	74.4	0.07	Low
	Admission to hospital-severe subgroup	0	0	0	0	0	OR = 0.10	[0.04,0.27]	0	<0.001	High
	PEFR	0	−1^②^	0	−1^④^	0	WMD = 29.0	[−3.00,62.00]	58.8	0.08	Low
	PEFR-severe subgroup	0	0	0	0	0	WMD = 52.0	[27.0,78.0]	0	<0.001	High
	FEV1%	0	−1^②^	0	−1^④^	0	WMD = 4.0	[−2.00,11.00]	73.2	0.18	Low
	FEV1%-severe subgroup	0	0	0	0	0	WMD = 10.0	[4.00,16.00]	0	0.002	High
Cheuk et al. (32)	Hospitalization	0	0	0	0	−1^⑤^	OR = 0.290	[0.143,0.589]	45.3	0.13	Medium
	PEFR <60% predicted	0	0	0	0	−1^⑤^	OR = 0.155	[0.057,0.422]	0	0.97	Medium
	Percentage change of PEFR	0	−1^②^	0	−1^④^	−1^⑤^	MD = 8.58	[0.94,16.22]	96.7	<0.0001	Extremely low
	Clinical symptom score	0	−1^②^	0	−1^④^	−1^⑤^	MD = 1.33	[0.31,2.36]	92.1	0.0001	Extremely low
Blitz et al. (33)	Pulmonary function tests	−1^①^	0	0	−1^④^	−1^⑤^	SMD = 0.37	[0.10, 0.63]	0	0.006	Extremely low
	Hospital admission	−1^①^	0	0	−1^④^	−1^⑤^	RR = 0.69	[0.42, 1.12]	0	0.13	Extremely low
	Mild–moderate adverse events	−1^①^	0	0	−1^④^	−1^⑤^	OR = 0.56	[0.21, 1.45]	0	0.23	Extremely low
Lu and Zhou (34)	Pulmonary function	−1^①^	0	0	0	0	SMD = 0.05	[0.28,0.83]	0	<0.001	Medium
	Risk of hospital admission	−1^①^	−1^②^	0	−1^④^	0	RR = 0.64	[0.38,1.08]	N/A	0.096	Extremely low
Powell et al. (35)	FEV1	−1^①^	−1^②^	0	0	0	SMD = 0.23	[−0.27,0.74]	66	0.36	Low
	PEFR	−1^①^	0	0	−1^④^	0	MD = 7.07	[−11.69,25.84]	26	0.46	Low
	Hospital admission	−1^①^	0	0	−1^④^	0	RR = 0.76	[0.49,1.16]	0	0.20	Low
	Serious adverse events	−1^①^	0	0	0	0	RD = 0.00	[−0.03,0.03]	0	1.0	Medium
	Mild–moderate adverse events	−1^①^	0	0	−1^④^	0	RD = -0.03	[−0.14,0.08]	0	0.62	Low
Kew et al. (36)	Hospital admissions	0	0	0	0	0	OR = 0.75	[0.60,0.92]	28	0.18	high
	Length of hospital stay	0	−1^②^	0	0	0	MD = -0.03	[−0.33,0.27]	53	0.07	Medium
	Readmission	0	0	0	−1^④^	0	OR = 2.30	[0.66,7.99]	0	0.19	Medium
	Heart rate	0	−1^②^	0	0	0	MD = -2.37	[−4.13,-0.61]	78	0.008	Medium
	Respiratory rate	0	0	0	−1^④^	0	MD = -0.28	[−0.77,0.20]	1	0.25	Medium
	Systolic blood pressure	0	−1^②^	0	0	0	MD = 0.08	[−1.89,2.05]	51	0.94	Medium
	FEV1 (% predicted)	0	0	0	0	0	MD = 4.41	[1.75,7.06]	14	0.001	High
	PEFR (% predicted)	0	0	0	0	0	MD = 4.78	[2.14,7.43]	45	0.0004	High
	PEFR (L/min)	0	−1^②^	0	0	0	MD = 17.4	[8.64,26.17]	50	0.0001	Medium
	Borg Dyspnea Scale	0	0	0	0	0	MD = -0.22	[−0.55,0.12]	0	0.82	High
Wang et al. (37)	Percentage increase in PEFR from baseline (10 min)	−1^①^	−1^②^	0	0	0	SMD = -0.75	[−1.29,-0.22]	72	0.005	Low
	Percentage increase in PEFR from baseline (20 min)	−1^①^	−1^②^	0	0	0	SMD = -0.46	[−0.72,-0.20]	47	0.006	Low
	FEV1% predicted (10 min)	−1^①^	−1^②^	0	0	0	SMD = -0.03	[−0.52,0.46]	74	0.89	Low
	FEV1% predicted (20 min)	−1^①^	−1^②^	0	0	0	SMD = -0.04	[−0.29,0.37]	47	0.81	Low
	PEFR% predicted (10 min)	−1^①^	0	0	0	0	SMD = -0.02	[−0.23,0.19]	0	0.83	Medium
	PEFR% predicted (20 min)	−1^①^	0	0	0	0	SMD = -0.05	[−0.31,0.42]	60	0.78	Medium
Ma and Zhao (38)	Remission rate of clinical signs	−1^①^	0	0	0	−1^⑤^	OR = 5.50	[3.73,8.11]	10	0.97	Low
	Recurrence rate of asthma	−1^①^	0	0	0	0	OR = 0.26	[0.15,0.43]	0	0.80	Medium
	PEFR	−1^①^	−1^②^	0	−1^④^	0	SMD = 0.85	[0.11,1.59]	100	<0.01	Extremely low
	FEV1% (percentage of predicted)	−1^①^	−1^②^	0	−1^④^	0	SMD = 7.65	[2.60,12.71]	100	<0.01	Extremely low
	FEV1/Forced Vital Capacity%	−1^①^	−1^②^	0	−1^④^	0	SMD = 6.31	[5.47,7.14]	100	<0.01	Extremely low
	Adverse drug reactions	−1^①^	0	0	−1^④^	0	OR = 4.43	[1.87,10.49]	0	0.75	Low
Ling et al. (39)	Pulmonary function-acute asthma	−1^①^	−1^②^	0	−1^④^	−1^⑤^	SMD = 0.39	[−0.03,0.82]	90	0.07	Extremely low
	Pulmonary function-stable asthma	−1^①^	−1^②^	0	−1^④^	−1^⑤^	SMD = 1.48	[−0.14,3.11]	N/A	0.07	Extremely low
	Hospital admission	0	0	0	−1^④^	−1^⑤^	RR = 0.72	[0.52,1.00]	49	0.05	Low
	Adverse events	−1^①^	0	0	−1^④^	−1^⑤^	RR = 1.15	[0.88,1.52]	14	0.31	Extremely low
Knightly et al. (40)	Pulmonary function (% predicted FEV1)	−1^①^	−1^②^	0	−1^④^	0	MD = 3.28	[1.06,5.49]	64	0.0038	Extremely low
	Pulmonary function (% predicted PEFR)	0	−1^②^	0	0	0	MD = 0.05	[−2.33,2.42]	67	0.97	Medium
	Clinical severity scores	−1^①^	−1^②^	0	0	0	SMD = 0.01	[−0.11,0.12]	83	0.92	Low
	Admission at first presentation	0	−1^②^	0	0	0	RR = 0.95	[0.91,1.00]	52	0.049	Medium
	Readmission	0	0	0	0	0	RR = 1.80	[0.84,3.87]	37	0.13	High
	Serious adverse events	0	0	0	−1^④^	0	RD = -0.03	[−0.06,0.00]	0	0.023	Medium
	Any adverse events	0	0	0	0	0	RD = 0.01	[−0.03,0.05]	0	0.77	High
Kassab et al. (41)	FEV1	−1^①^	0	0	−1^④^	0	SMD = 0.15	[0.00,0.31]	0	0.06	Low
	Vital Signs	−1^①^	−1^②^	0	−1^④^	0	SMD = -0.11	[−0.27,0.04]	68	0.16	Extremely low
	Asthma Severity Score	−1^①^	−1^②^	0	−1^④^	0	SMD = 0.22	[0.01,0.44]	88	0.04	Extremely low
	Peak Expiratory Flow Rate	−1^①^	−1^②^	0	−1^④^	0	SMD = 2.02	[0.83,3.21]	98	<0.001	Extremely low
	Modified Pulmonary Index Score	−1^①^	0	0	−1^④^	0	MD = 0.30	[−0.25,1.01]	N/A	0.24	Low
Zhang et al. (42)	Clinical total effective rate	−1^①^	0	0	0	0	RR = 1.15	[1.09,1.20]	16	<0.00001	Medium
	FEV1	−1^①^	0	0	0	0	MD = 0.10	[−0.02,0.18]	0	0.02	Medium
	PEFR	−1^①^	0	0	0	0	MD = 0.38	[0.26,0.49]	40	<0.00001	Medium
	Adverse reaction rate	−1^①^	0	0	−1^④^	0	RR = 2.13	[0.79,5.78]	0	0.14	Low
Ambrozej et al. (43)	Admission rate	−1^①^	−1^②^	0	−1^④^	0	OR = 0.15	[0.03,0.73]	67	<0.05	Extremely low
	PEFR	−1^①^	0	0	−1^④^	0	MD = 26.77	[18.41,54.79]	43	<0.05	Low
	Length of hospital stay	−1^①^	−1^②^	0	−1^④^	0	MD = -6.16	[−17.49,5.17]	95	>0.05	Extremely low
Kumar et al. (44)	Composite Asthma Severity Score	0	0	0	−1^④^	0	SMD = -0.09	[−0.20,0.02]	21	0.10	Medium
	Need for hospitalization	0	0	0	−1^④^	0	RR = 0.92	[0.79,1.06]	N/A	0.24	Medium
	Need for ICU admission	0	0	0	−1^④^	0	RR = 1.29	[0.75,2.20]	N/A	0.36	Medium
	Discharged by 24 h	0	0	0	0	0	RR = 1.02	[0.95,1.10]	N/A	0.60	High
	Length of hospital stay	0	0	0	−1^④^	0	MD = -4.60	[−10.0,0.80]	N/A	0.10	Medium
	PEFR	0	0	0	−1^④^	0	MD = 19.30	[8.90,29.80]	0	<0.01	Medium
	FEV1	0	0	0	−1^④^	0	MD = 8.10	[−3.00,19.20]	N/A	0.15	Medium
	Heart rate	0	0	0	−1^④^	0	MD = -2.70	[−8.20,2.70]	N/A	0.32	Medium
	Respiratory rate	0	0	0	0	0	MD = -0.90	[−1.40,0.40]	N/A	<0.01	High
	Oxygen saturation	0	0	0	−1^④^	0	MD = 1.20	[−3.50,5.80]	N/A	0.63	Medium
	Any adverse event	0	0	0	−1^④^	0	RR = 1.45	[0.63,3.34]	N/A	0.38	Medium
Cunha et al. (45)	Respiratory rate	0	0	0	0	0	MD = -0.70	[−1.24,-0.15]	6	0.01	High
	PEFR	0	0	0	0	0	MD = 5.33	[4.75,5.90]	24	<0.01	High
	FEV1	0	0	0	−1^④^	0	MD = 1.82	[−1.89,5.53]	0	0.34	Medium
	Peripheral O₂ saturation	0	−1^②^	0	0	0	MD = 0.32	[−0.29,0.94]	83	0.31	Medium
	Heart rate	0	−1^②^	0	0	0	MD = -4.06	[−8.17,0.05]	74	0.05	Medium
	Asthma severity scores	0	−1^②^	−1^③^	0	0	SMD = -0.04	[−0.28,0.20]	79	0.75	low
	Need for intravenous bronchodilator use	0	0	0	−1^④^	0	RR = 0.86	[0.70,1.06]	0	0.15	Medium
	Hospitalization events	0	0	0	−1^④^	0	RR = 0.92	[0.79,1.06]	6	0.80	Medium
Zhong et al. (46)	Total effective rate	−1^①^	0	0	0	0	RR = 1.11	[1.03,1.20]	0	0.008	Medium
	PEFR	−1^①^	−1^②^	0	0	−1^⑤^	WMD = 0.70	[0.24,1.15]	94.1	0.003	Extremely low
	FEV1	−1^①^	−1^②^	0	0	−1^⑤^	WMD = 0.48	[0.29,0.68]	85.6	<0.001	Extremely low
	Forced Vital Capacity	−1^①^	−1^②^	0	0	−1^⑤^	WMD = 0.72	[0.47,0.97]	79.0	<0.001	Extremely low
	Incidence of adverse reactions	−1^①^	−1^②^	0	−1^④^	−1^⑤^	RR = 0.51	[0.17,1.55]	67.4	0.419	Extremely low
Hamud et al. (47)	Asthma severity score	−1^①^	−1^②^	0	−1^④^	0	MD = -0.18	[−1.35,0.98]	2	>0.05	Extremely low
	Hospitalization rate	−1^①^	0	0	−1^④^	0	RR = 0.70	[0.54,0.90]	7	<0.05	Low
	Invasive ventilation	−1^①^	−1^②^	0	-1^④^	0	RR = 0.35	[0.11,1.17]	0	>0.05	Extremely low
	Non-invasive ventilation	0	-1^②^	0	-1^④^	0	RR = 0.17	[0.05,0.54]	N/A	0.003	Low
	PICU admission	0	0	0	-1^④^	0	RR = 0.58	[0.15,2.22]	N/A	0.43	Medium
	Hospital stay	-1^①^	-1^②^	0	-1^④^	0	MD = -78.86	[−200.37,42.66]	99	>0.05	Extremely low
	PEFR improvement	0	0	0	-1^④^	0	MD = 36.60	[33.28,39.92]	N/A	<0.00001	Medium

① Methodological quality of included studies was low, with biases in randomization, allocation concealment, and blinding; ② The heterogeneity was large and low confidence interval overlap; ③ The population was not broadly representative; ④ Small sample size, 95% confidence intervals include null values; ⑤ Few studies were included, the funnel plot was not symmetrical, Egger’s test found that publication bias or results were positive and there was no publication bias evaluation.

### 3.8 Quantitative analysis

#### 3.8.1 Admission rate

**Figure 4:**
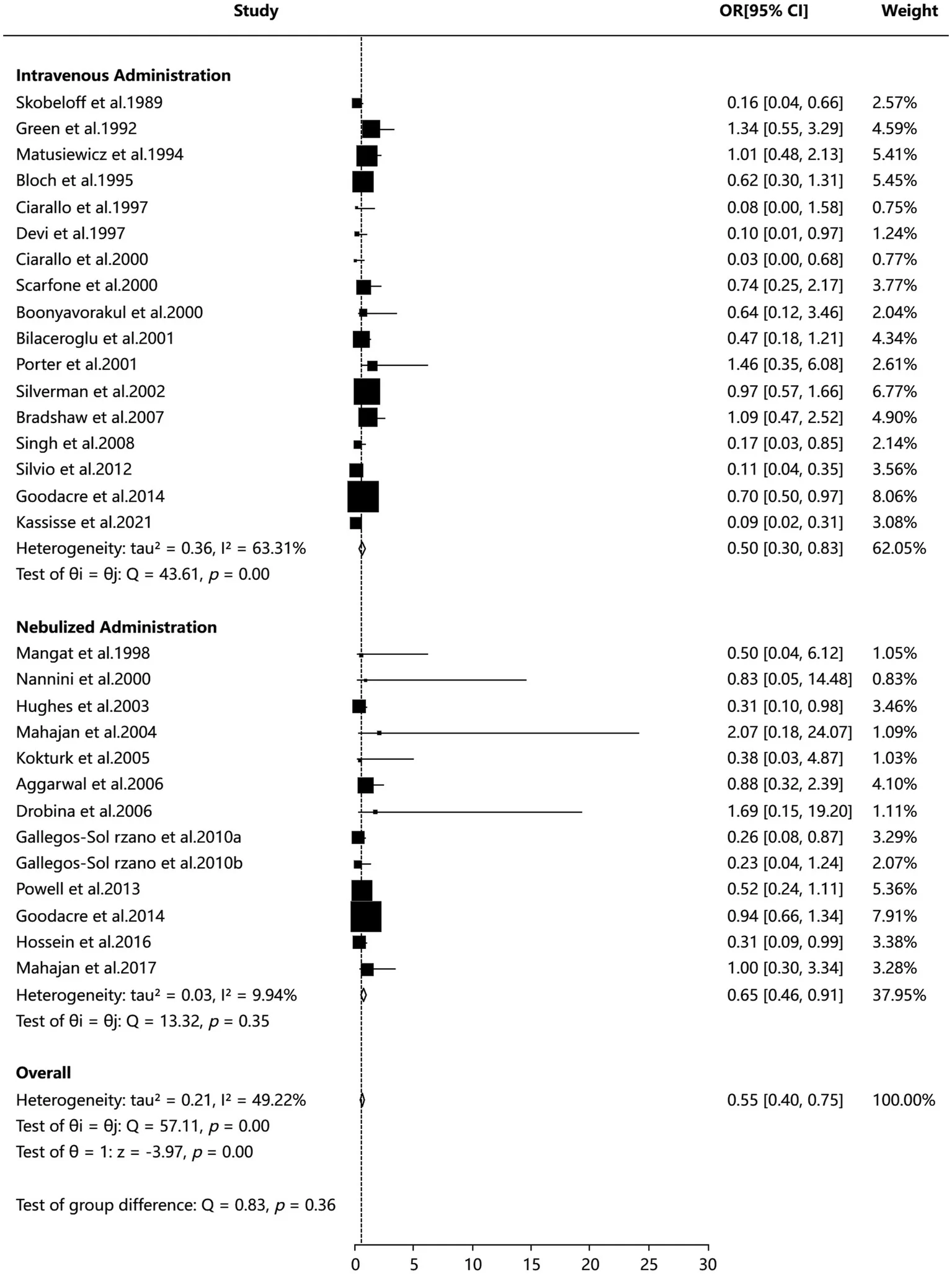
Meta-analysis of admission rate.

In total, 12 of the included SRs/MAs (30–36, 39, 43–45, 47) reported quantitative data on hospitalization rates. Quantitative analysis using a random-effects model showed that intravenous MgSO_4_ treatment was associated with a lower hospitalization rate than the control group [OR = 0.50, 95% CI (0.30, 0.83), *p* < 0.05]. This finding indicates that intravenous MgSO_4_ significantly reduces the risk of hospitalization in asthma patients. However, considerable heterogeneity was observed across studies (*I*^2^ = 63.31%). Although sensitivity analyses and subgroup analyses were conducted, the sources of heterogeneity remain unclear. Nebulized MgSO_4_ may contribute to a reduced hospitalization risk in asthma patients [OR = 0.65, 95% CI (0.46, 0.91)], with low heterogeneity among studies (*I*^2^ = 9.94%). The between-group difference did not reach statistical significance (*p* > 0.05), and further research is needed to clarify its clinical relevance. The effect size of intravenous MgSO_4_ appeared numerically superior to that of nebulized MgSO_4_ in reducing asthma hospitalization rates, though the between-group difference was not statistically significant (*p* > 0.05). This observation may be attributable to variations in study design, patient populations, or dosing regimens. Detailed results are presented in Figure 4.

#### 3.8.2 Percent predicted FEV1

**Figure 5:**
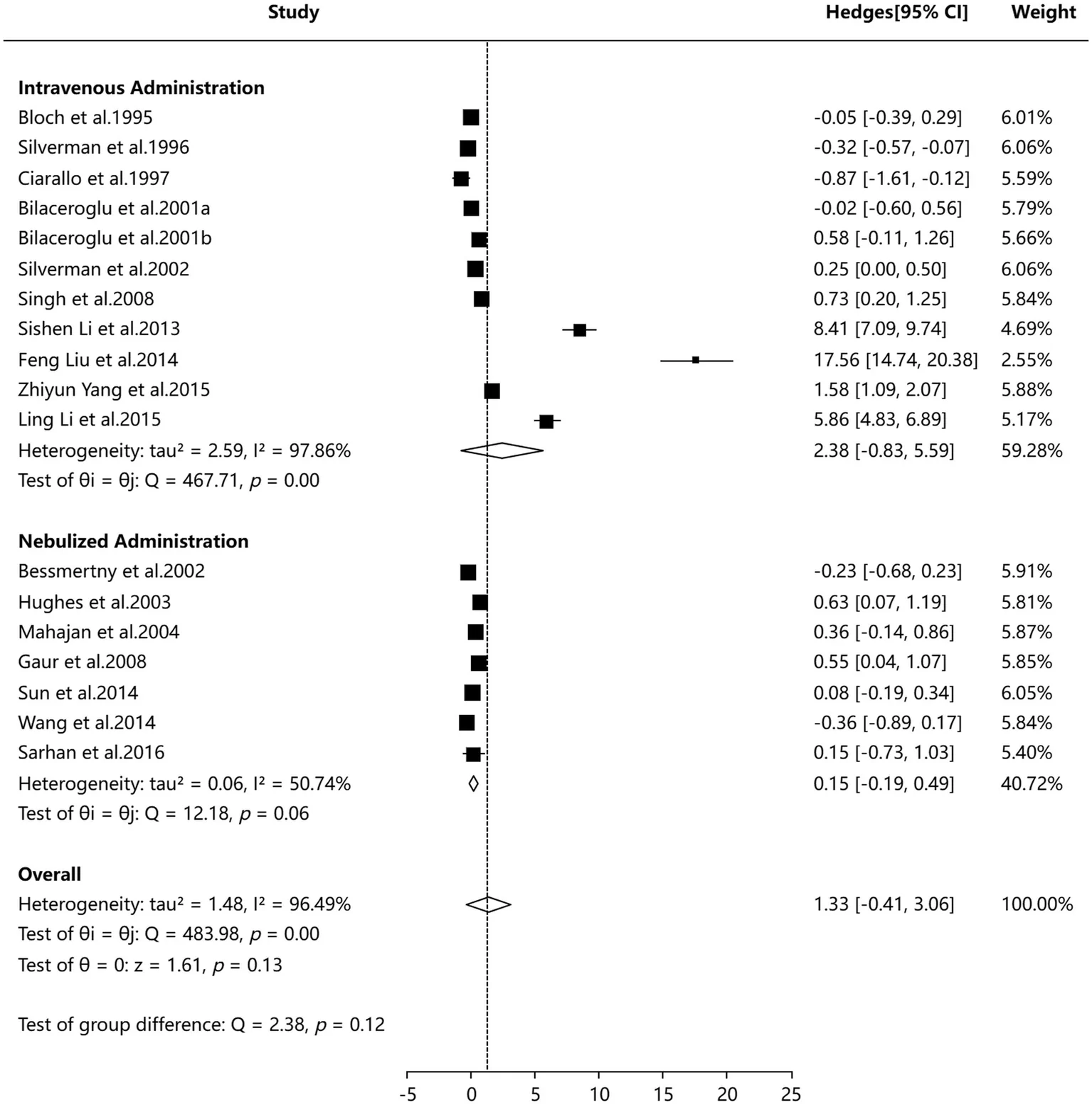
Meta-analysis of percent predicted FEV1.

In total, 10 of the included SRs/MAs (30, 33, 35–38, 40–42, 44) reported quantitative data on the percentage of FEV1. Random-effects meta-analysis showed that intravenous MgSO_4_ did not significantly change FEV1 percentage in asthma patients [SMD = 2.38, 95% CI (−0.83, 5.59), *p* > 0.05]. However, substantial heterogeneity was observed across studies (*I*^2^ = 97.86%). Similarly, nebulized MgSO_4_ did not show a significant effect on the percentage of FEV1 [SMD = 0.15, 95% CI (−0.19, 0.49), *p* > 0.05, *I*^2^ = 50.74%]. Although the effect size for intravenous administration was numerically larger than that for nebulized administration, the difference between the two groups was not statistically significant (*p* > 0.05). Detailed results are presented in Figure 5.

#### 3.8.3 Absolute PEFR

**Figure 6:**
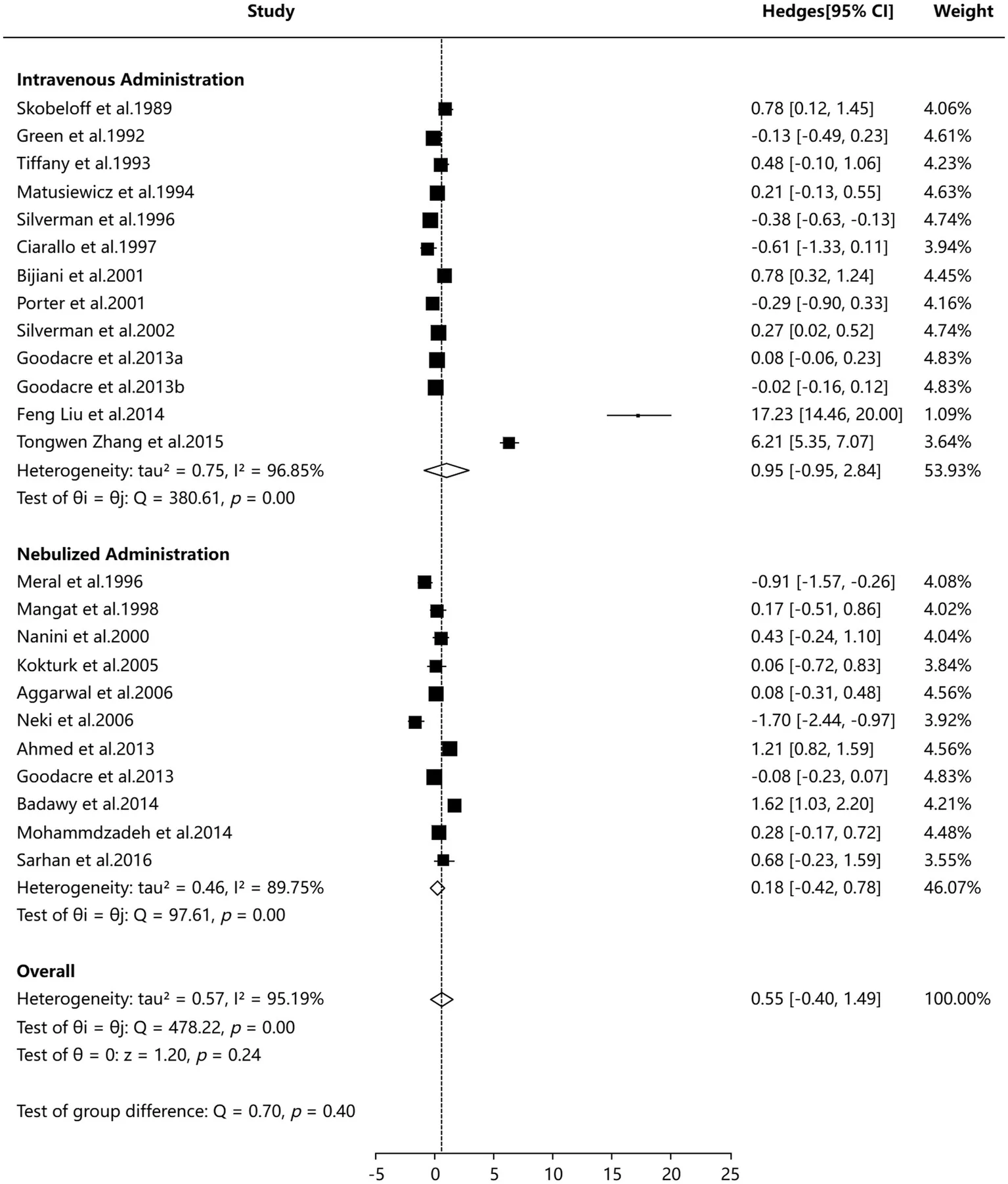
Meta-analysis of absolute PEFR.

A total of 10 of the included SRs/MAs (30, 33–38, 40–42) reported quantitative data on absolute PEFR. Random-effects meta-analysis showed that intravenous MgSO_4_ was associated with a higher absolute PEFR in asthma patients than the control group [SMD = 0.95, 95% CI (−0.95, 2.84)], but this difference was not statistically significant (*p* > 0.05). Substantial heterogeneity was observed across studies (*I*^2^ = 96.85%). Similarly, nebulized MgSO_4_ showed no significant difference in absolute PEFR versus the control group [SMD = 0.18, 95% CI (−0.42, 0.78), *p* > 0.05, *I*^2^ = 89.75%]. Although the effect estimate for intravenous administration was numerically higher than that for nebulized inhalation, the between-group difference was not statistically significant (*p* > 0.05). Detailed results are presented in Figure 6.

#### 3.8.4 Vital signs

**Figure 7:**
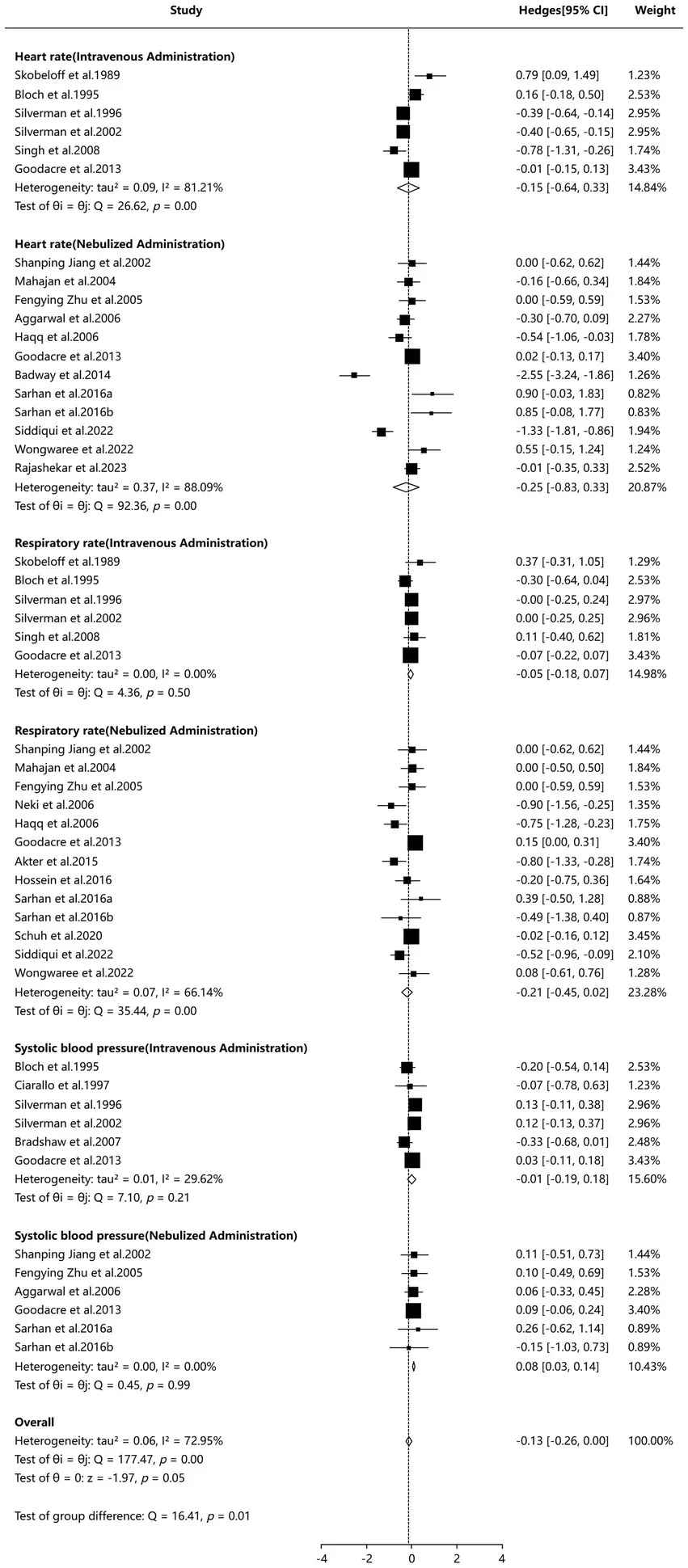
Meta-analysis of vital signs.

In total, 10 of the included SRs/MAs (30, 31, 33–36, 40, 41, 44, 45) reported quantitative data on vital signs. Random-effects meta-analysis showed that intravenous MgSO_4_ had no statistically significant effects on heart rate, respiratory rate, or systolic blood pressure in asthma patients. The specific results were as follows: heart rate [SMD = −0.15, 95% CI (−0.64, 0.33), *p* > 0.05, *I*^2^ = 81.21%], respiratory rate [SMD = −0.05, 95% CI (−0.18, 0.07), *p* > 0.05, *I*^2^ = 0.00%], and systolic blood pressure [SMD = −0.01, 95% CI (−0.19, 0.18), *p* > 0.05, *I*^2^ = 29.62%]. Similarly, nebulized MgSO_4_ showed no significant effect on heart rate [SMD = −0.25, 95% CI (−0.83, 0.33), *p* > 0.05, *I*^2^ = 88.09%]. Its effect on respiratory rate was of borderline statistical significance [SMD = −0.21, 95% CI (−0.45, 0.02), P ≈ 0.05, *I*^2^ = 66.14%], while it significantly increased systolic blood pressure [SMD = 0.08, 95% CI (0.03, 0.14), *p* < 0.05, *I*^2^ = 0.00%]. Between-group comparison tests revealed statistically significant differences in the effects of the two administration routes. Overall, intravenous administration did not demonstrate significant regulatory effects on the above vital sign indicators. In contrast, nebulized administration not only significantly elevated systolic blood pressure but also showed a potential trend of influence on respiratory rate, indicating differential impacts of the two administration routes on vital signs in asthma patients. Nebulized inhalation exhibited a distinct effect profile compared with intravenous administration in certain indicators. Detailed results are presented in Figure 7.

#### 3.8.5 Adverse events

**Figure 8:**
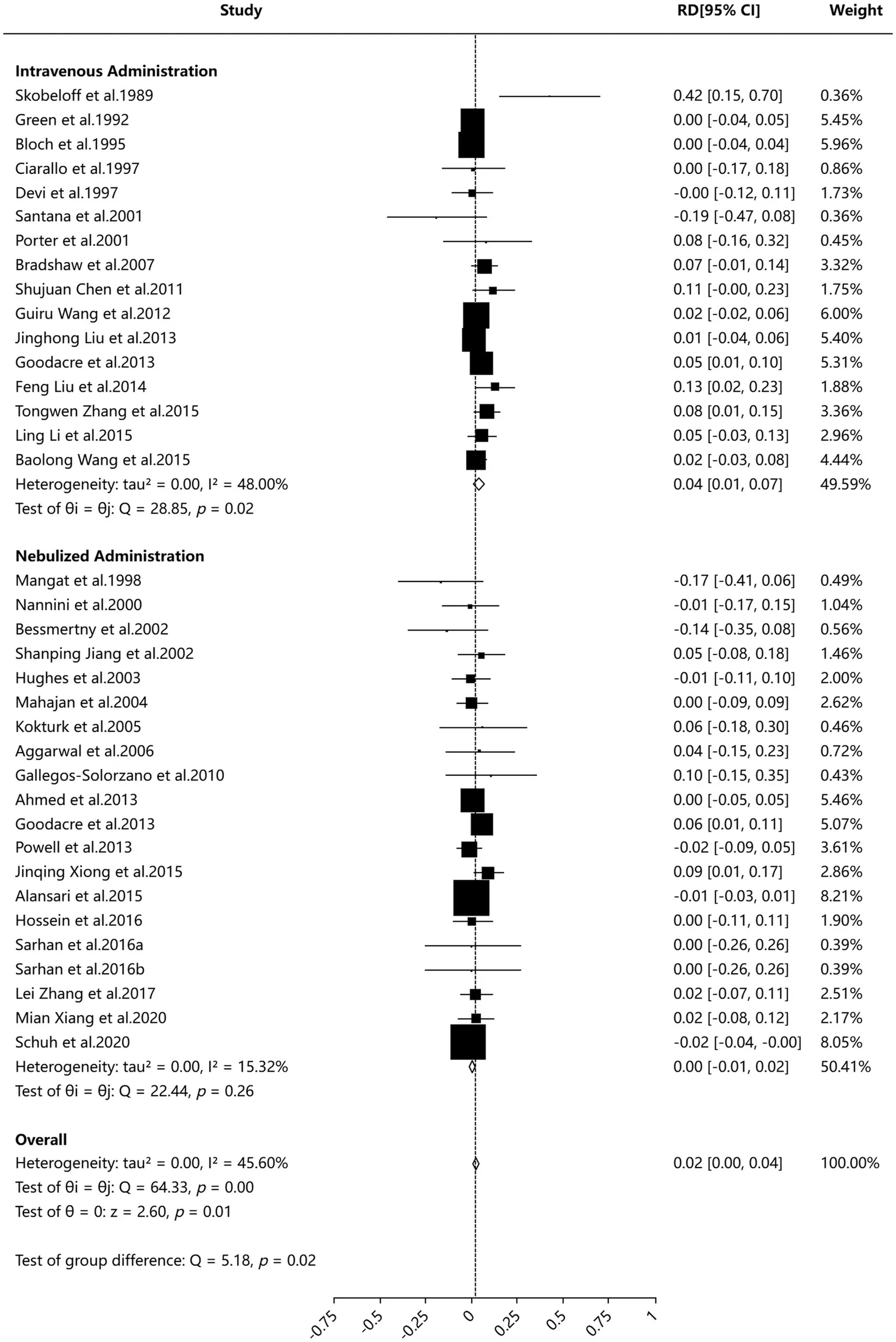
Meta-analysis of adverse events.

In total, 14 of the included SRs/MAs (30, 31, 35–40, 42–47) reported quantitative data on adverse events. Random-effects meta-analysis showed that intravenous MgSO_4_ was associated with a significantly lower incidence of adverse events than the control group in asthma patients [OR = 0.04, 95% CI (0.01, 0.07), *p* = 0.02]. Moderate heterogeneity was observed among studies (*I*^2^ = 48.00%). Although sensitivity and subgroup analyses were conducted, the source of heterogeneity remained unclear. In contrast, nebulized MgSO_4_ also showed a lower incidence of adverse events versus the control group [OR = 0.00, 95% CI (−0.01, 0.02)], but this result was not statistically significant (*p* = 0.26), with low heterogeneity (*I*^2^ = 15.32%). Comparing intravenous versus nebulized administration, the effect estimate for intravenous MgSO_4_ was higher than that for nebulized MgSO_4_, but the between-group difference was statistically significant (Q = 5.18, *p* = 0.02). This lack of significant difference may be attributable to variations in study design, patient populations, or dosing regimens. Detailed results are presented in Figure 8.

### 3.9 Qualitative analysis

#### 3.9.1 Critical care

In total, 15 of the included SRs/MAs (36, 40, 43, 44, 47) evaluated intensive care treatment as an outcome measure. These studies collectively indicated that current evidence does not definitively demonstrate that either intravenous or nebulized MgSO_4_ significantly reduces overall intensive care unit (including ICU/HDU/PICU) admission rates in adults or children with asthma. However, the evidence is generally limited by the small number of studies, low event rates, and low quality of evidence. Intravenous MgSO_4_ (36, 43, 47) showed potential benefit in several important secondary intensive care outcomes, such as reducing the need for mechanical ventilation. Given its widely recognized safety profile, it is often considered in guidelines as a second-line therapy for severe asthma. In contrast, nebulized MgSO_4_ (40, 44) demonstrated no significant advantage in primary intensive care-related outcomes. Its role appears to lean more toward potential improvement in pulmonary function, with unclear effects on critical care outcomes. Consequently, it is not recommended as a routine second-line therapy. Nebulized MgSO_4_ may be more suitable as a preventive treatment rather than an intervention for critical asthma episodes.

#### 3.9.2 Therapy duration

In total, 7 of the included SRs/MAs (30, 35, 36, 38, 43, 44, 47) evaluated treatment duration as an outcome measure. A total of 4 studies (30, 36, 38, 47) consistently indicated that intravenous MgSO_4_ did not significantly reduce overall emergency department treatment time. However, one study (38) suggested a potential reduction in time to symptom relief or ED length of stay in severe asthma subgroups or when used as combination therapy, though this evidence remains limited and requires cautious interpretation. The same study (38) also demonstrated that intravenous MgSO_4_ effectively accelerated the resolution of clinical symptoms such as cough and wheezing, potentially shortening the duration of acute-phase treatment. One study (43) proposed that early combination with first-line therapy might optimize emergency department workflows. In contrast, two studies (35, 44) found that nebulized MgSO_4_ showed no significant improvement in treatment duration, thus not supporting its use as a routine second-line therapy.

## 4 Discussion

### 4.1 Comparison between intravenous and nebulized magnesium sulfate

The core mechanism of intravenous MgSO_4_ in treating bronchial asthma lies in magnesium ions acting as physiological calcium antagonists (48), which directly relax constricted bronchial smooth muscle (49). Simultaneously, it mitigates airway spasm and inflammation through multiple pathways: by inhibiting acetylcholine release and reducing cholinergic nerve tension (50), as well as by stabilizing mast cell membranes to diminish the release of inflammatory mediators such as histamine and leukotrienes (51, 52), thereby alleviating airway narrowing and inflammatory responses (52). The findings suggest that intravenous MgSO_4_ may confer clinical benefit in acute asthma management, primarily reflected in reduced admission risk—an outcome closely tied to short-term prognosis and healthcare resource utilization—implying that early intravenous administration may modulate pathophysiological processes, potentially attenuating acute inflammatory cascades or bronchospasm progression. No significant improvements were observed in FEV1% or absolute PEFV, possibly because the drug’s primary targets involve systemic inflammation rather than direct relief of airway obstruction. Notably, among severe secondary outcomes, intravenous MgSO_4_ may reduce mechanical ventilation requirements; coupled with a lower adverse event rate versus controls, these findings support its safety and potential efficacy as a second-line therapy for severe asthma. Some studies suggest potential reductions in emergency department length of stay in severe subgroups or combination therapy contexts, though overall evidence for treatment duration benefit remains limited and warrants cautious interpretation. In summary, given decreased admission risk, manageable adverse effects, and potential supportive effects in severe cases, intravenous MgSO_4_ represents a viable intervention option for acute severe asthma, particularly in scenarios aiming to curb disease progression and alleviate healthcare burden—though higher-quality studies are needed for confirmation. However, despite statistically significant benefits in reducing admission and adverse events, the evidence for these outcomes was only moderate in quality, downgraded due to population heterogeneity, inconsistent dosing regimens, and substantial heterogeneity. Statistical significance alone does not support routine use in the general population; rather, intravenous MgSO_4_ is recommended only as an add-on second-line therapy for patients who have failed first-line treatment in severe settings. This recommendation strength is determined by GRADE evidence quality, not purely by statistical results.

The mechanism of nebulized MgSO_4_ in the treatment of bronchial asthma primarily relies on the local hypertonic environment it creates. This promotes the efflux of edema fluid from the airway mucosa via an osmotic gradient (53), thereby reducing edema and increasing airway diameter (52). Simultaneously, it stimulates mucosal water secretion to dilute sputum, facilitating its expulsion and relieving obstruction. The hypertonic state may also relax smooth muscle by altering the extracellular ionic environment (49), indirectly antagonizing calcium-mediated contraction (48). These effects are complemented by the local action of magnesium ions in antagonizing calcium and stabilizing mast cells, collectively contributing to bronchodilation and anti-inflammatory effects. Preliminary findings suggest that nebulized MgSO_4_ may exert predominantly local effects with potential prophylactic value. Its admission reduction effect appears weaker than that of intravenous administration, with overall safety being acceptable—no significant between-group difference in adverse event rates was observed, possibly attributable to limited systemic absorption via airway delivery. No significant improvements in pulmonary function were detected, suggesting limited direct bronchodilatory or ventilatory effects. Vital sign analyses showed trends toward slight respiratory rate modulation and modest systolic blood pressure elevation, potentially via mild local anti-inflammatory or weak bronchodilator actions marginally affecting respiratory mechanics. Current data do not support benefits in intensive care endpoints (e.g., ICU admission) or overall treatment duration reduction; thus, evidence does not support routine second-line use. However, secondary outcome data hint at potential utility in mild exacerbation intervention or prevention through modest stabilization and pulmonary function improvement. Given its predominantly local action and minimal systemic effects, nebulized MgSO_4_ may suit stable-phase adjuvant therapy or patients requiring avoidance of high systemic drug exposure, offering preliminary reference for individualized asthma regimens. In conclusion, its clinical value warrants case-by-case assessment based on disease stage and patient characteristics, with further high-quality studies needed to confirm its preventive and mild-case management potential. Although nebulized MgSO_4_ showed statistically significant numeric improvements in pulmonary function and respiratory rate, over 70% of nebulization-related outcomes were based on low/very low quality evidence. Statistical differences merely indicate minor local physiological changes, undermined by high heterogeneity, publication bias, and airway differences between children and adults—rendering evidence insufficiently credible. Therefore, routine nebulized MgSO_4_ use is not supported; only a weak recommendation may be made for trial after standard therapy failure.

### 4.2 Evaluation of literature quality

This study underwent a rigorous quality assessment, which revealed several deficiencies in both methodological and reporting dimensions. Methodologically, the review failed to clearly state whether the review methodology was pre-specified prior to conduct or to provide a rationale for any significant deviations from the protocol, thereby compromising the transparency and reproducibility of the research process. It did not provide a list of excluded studies along with the reasons for their exclusion, raising concerns regarding the comprehensiveness and objectivity of the study. The absence of reported funding sources for the included studies impedes the assessment of potential conflicts of interest. Furthermore, the authors did not assess the potential impact of individual study risk of bias on the pooled results, nor did they adequately investigate publication bias or its potential influence on the review’s conclusions. These omissions substantially undermine the reliability and accuracy of the findings. Regarding reporting quality, the review did not describe the specific methods used to assess the certainty of outcome evidence, nor did it provide graded certainty ratings for each outcome. This limits the ability to judge the credibility of the results. Regarding registration and protocol, it failed to provide the review registration information, access details for the review protocol, or an account of any amendments made to the registered/protocol information, which hinders the traceability and verification of the study. Additionally, the availability and access details for data, code, and related materials were not reported, limiting the potential for independent verification and secondary use by other researchers. The cumulative effect of these methodological and reporting deficiencies collectively diminishes the overall quality and trustworthiness of this study. Future research of a similar nature should address these shortcomings systematically to enhance the quality standards and scientific value of SRs/MAs.

In future, SRs/MAs researchers in this field should prioritize the optimization of methodological and reporting quality. In terms of methodological refinement, it is essential to predefine the review methodology prior to study initiation and to systematically describe any deviations from the predefined protocol along with their underlying reasons in the final report. This approach can significantly enhance the transparency and credibility of the research. A complete list of excluded studies and the rationale for their exclusion should be provided to ensure comprehensive coverage. Additionally, detailed disclosure of funding sources for included studies is necessary to mitigate potential bias arising from conflicts of interest that may compromise the objectivity of the findings. When conducting meta-analyses, it is crucial to systematically evaluate the potential impact of individual study bias on the pooled results. Furthermore, thorough assessment of publication bias and its possible influence on the review’s conclusions should be undertaken to reinforce the scientific rigor and validity of the study. Regarding the improvement of reporting quality, strengthening the evaluation of evidence certainty is imperative. This includes not only a clear description of the specific methods used to determine the certainty of outcomes but also a graded assessment of the certainty of evidence for each outcome, thereby enabling readers to accurately interpret the reliability of the findings. Standardized study registration and protocol management should be implemented, with complete provision of review registration details, access routes to the review protocol, and explicit explanations of any modifications made to the registered or protocol information. These measures will enhance the standardization and traceability of the research. Finally, emphasis should be placed on the accessibility of data, code, and related materials. The scope of publicly available content and specific access pathways should be reported to support reproducibility and promote academic transparency.

This study used the GRADE tool to grade evidence for all 104 outcome indicators. Only 13 were rated as high-quality. Evidence for nebulized MgSO_4_ was predominantly low or very low quality (over 70% of all nebulization-related outcomes). Downgrading factors included risk of bias, high heterogeneity, small sample sizes, and publication bias. Evidence certainty directly limits the generalizability of clinical conclusions. Statistically significant positive results supported by high-quality evidence can be translated into strong recommendations. Moderate-quality evidence supports only cautious, limited recommendations. For low/very low-quality evidence, even when meta-analyses show statistical trends, no definitive clinical benefit can be concluded. This is especially pertinent for nebulized magnesium sulfate, where many indicators show only numerical trends without high-certainty support, warranting extreme caution in clinical use. Most current reviews report only pooled effect sizes without GRADE-based stratification of confidence, potentially leading to overinterpretation of potential efficacy. This study addresses this gap through evidence grading.

Additionally, the overall ROBIS risk of bias across all included studies was rated low in this review. Although this appears inconsistent with the AMSTAR-2 and PRISMA 2020 results, the discrepancy arises from fundamentally different evaluation frameworks: ROBIS assesses systematic bias in study conduct, AMSTAR-2 evaluates methodological completeness and rigor, and PRISMA 2020 examines reporting transparency. Low AMSTAR-2 scores or PRISMA 2020 deficiencies indicate inadequate documentation, not methodological flaws that distort treatment effects. Given the non-overlapping dimensions, the ROBIS low-risk conclusion remains valid, and no inherent contradiction exists.

### 4.3 Sources and interpretation of heterogeneity in bodies of evidence

In this study, upon re-evaluating 18 SRs/MAs, we observed significant or extremely high statistical heterogeneity in pooled analyses of several core outcomes (e.g., *I*^2^ = 63.31% for admission rate and *I*^2^ = 97.86% for FEV1% in the intravenous administration group). Although we applied random-effects models to partially mitigate this issue and conducted subgroup and sensitivity analyses based on potential methodological heterogeneity (e.g., study design and risk of bias), we could not fully trace the underlying sources. Prudent interpretation of these highly heterogeneous results is essential to avoid oversimplified conclusions. Below, we systematically explore potential sources of heterogeneity from both clinical and methodological perspectives:

(1) Age stratification: The original studies on intravenous magnesium sulfate covered both adults and children. Children differ from adults in bronchial smooth muscle physiology and renal magnesium clearance; thus, at equivalent doses, improvements in pulmonary function and reductions in admission risk vary significantly by age. Nebulized magnesium studies included a higher proportion of children, in whom smaller airway diameters lead to substantially different aerosol deposition efficiency compared with adults, directly increasing heterogeneity in pooled estimates. (2) Regimen differences: Intravenous dosing ranged from 1.2 to 4 g, with infusion durations varying widely from 20 min to 2 h. Nebulized magnesium was delivered as either isotonic or hypertonic solutions, with single nebulization sessions ranging from 5 to 20 min. These diverse regimens result in differing local/systemic magnesium exposures, leading to inconsistent bronchodilatory and anti-inflammatory effects. (3) Care settings: Emergency studies used short-term lung function and immediate admission as endpoints, whereas ICU/PICU studies used mechanical ventilation, PICU admission, and long-term recurrence. Inconsistent endpoints, follow-up durations, and admission criteria across settings further amplified effect-size heterogeneity. (4) Variation in intervention timing: Some studies administered it as a first-line therapy with concurrent medications in the emergency setting, while others used it as a second-line rescue after first-line failure. Early versus rescue interventions correspond to fundamentally different baseline bronchospasm severity and inflammatory burden, leading to marked differences in observed endpoint improvements. (5) Differences in combination therapy: Some original studies used β_2_-agonists alone as background therapy, whereas others added systemic corticosteroids, ipratropium bromide, or montelukast. Synergistic effects among multiple drugs may amplify or mask the independent effect of MgSO_4_, ultimately increasing the dispersion of pooled effect estimates. (6) Heterogeneity in acute asthma severity: The included studies lacked uniform criteria for enrolling mild, moderate, or severe acute exacerbations, resulting in mixed patient populations. Disease severity directly influences bronchospasm and inflammatory burden, altering the therapeutic threshold of MgSO_4_—a key confounder affecting the stability of pooled effect sizes.

### 4.4 Limitation

This study has several limitations. (1) The included SRs/MAs may be influenced by biases present in the original studies, over which we had no control, potentially affecting the overall results; (2) Despite efforts to maintain objectivity, some degree of subjective judgment by the researchers during the evaluation process is unavoidable and may have introduced bias; (3) Although this study performed quantitative synthesis, the comparison between intravenous and nebulized administration was indirect only, without head-to-head direct evidence. Coupled with varying degrees of heterogeneity across quantitative analyses, this precludes robust evidence-based conclusions; (4) The methodological quality of the included SRs/MAs was variable, outcome definitions were inconsistent across studies, and there may have been publication bias and language bias. Additionally, we were unable to perform patient-level analyses, and there was potential duplication of primary RCTs among the included reviews.

## 5 Conclusion

Guided by an umbrella review, this study synthesizes differential effects of intravenous versus nebulized MgSO_4_ in acute asthma and proposes stratified recommendations based on evidence credibility. For intravenous use, current evidence does not support routine application in all acute asthma patients, but may serve as a rescue option in select high-risk populations. Limited data suggest that in severe adults and children failing first-line therapy, intravenous MgSO_4_ may reduce admission and improve lung function. Future guidelines may thus shift from generalized to stratified use, positioning it as adjunctive therapy after standard care for severe asthma. For nebulized use, evidence shows marked age-related heterogeneity, precluding general use. In children, negative findings are consistent—routine use is not recommended; in adults, only marginal lung function trends (e.g., slight respiratory rate modulation) are observed, with limited credibility, insufficient for a firm recommendation. Based on low-to-moderate certainty evidence, nebulized MgSO_4_ is not advised as routine/early therapy, but may be tried as third-line after standard failure. Future research should address the following uncertainties: (1) For nebulized magnesium, abandon one-size-fits-all enrollment, precisely target subgroups most likely to benefit (e.g., specific age bands and severity strata), and explore local airway pharmacological mechanisms; (2) For intravenous magnesium, conduct phenotype-driven randomized controlled trials to clarify efficacy differences across eosinophilic/non-eosinophilic asthma and comorbidity profiles, and explore synergies with novel therapies such as biologics; (3) Perform head-to-head comparisons with standardized dosing regimens to define dose–response relationships and reduce clinical heterogeneity in the current evidence base.

## Data Availability

The original contributions presented in the study are included in the article/supplementary material, further inquiries can be directed to the corresponding author/s.

## Footnotes

1
https://www.spssau.com
